# Utilizing functional near-infrared spectroscopy for prediction of cognitive workload in noisy work environments

**DOI:** 10.1117/1.NPh.4.4.041406

**Published:** 2017-08-18

**Authors:** Ryan Gabbard, Mary Fendley, Irfaan A. Dar, Rik Warren, Nasser H. Kashou

**Affiliations:** a Wright State University, Biomedical, Industrial and Human Factors Engineering, Dayton, Ohio, United States; b Air Force Research Laboratory, Wright-Patterson Air Force Base, Dayton, Ohio, United States

**Keywords:** functional near-infrared spectroscopy, workload, prefrontal cortex, eye tracking, noise, anomaly detection

## Abstract

Occupational noise frequently occurs in the work environment in military intelligence, surveillance, and reconnaissance operations. This impacts cognitive performance by acting as a stressor, potentially interfering with the analysts’ decision-making process. We investigated the effects of different noise stimuli on analysts’ performance and workload in anomaly detection by simulating a noisy work environment. We utilized functional near-infrared spectroscopy (fNIRS) to quantify oxy-hemoglobin (HbO) and deoxy-hemoglobin concentration changes in the prefrontal cortex (PFC), as well as behavioral measures, which include eye tracking, reaction time, and accuracy rate. We hypothesized that noisy environments would have a negative effect on the participant in terms of anomaly detection performance due to the increase in workload, which would be reflected by an increase in PFC activity. We found that HbO for some of the channels analyzed were significantly different across noise types (p<0.05). Our results also indicated that HbO activation for short-intermittent noise stimuli was greater in the PFC compared to long-intermittent noises. These approaches using fNIRS in conjunction with an understanding of the impact on human analysts in anomaly detection could potentially lead to better performance by optimizing work environments.

## 1 Background

The ever-increasing rate of object identification through automation outpaces the ability of human analysts to process the captured images and has become an increasingly time-critical task.^1^^,^^2^ Analysts must be able to discern signals (targets) from noise that can lead to four possible decisions: hit, miss, false alarm, and correct rejection.^3^ Anomalies refer to finding outliers or patterns in a dataset that do not conform to expected or normal behavior.^4^ Certain factors increase the difficulty of timely anomaly detection due to their random and unexpected nature.^5^^,^^6^ Occupational noise is a frequent exposure in the analysts’ work environment in military intelligence, surveillance, and reconnaissance (ISR) operations. Noise impacts cognitive performance by acting as a stressor, which could interfere with the analysts’ decision-making process.^7^ It has been well-documented that occupational noise has a negative impact on job satisfaction.^8^^–^^11^ Previous studies have shown that noise stress has a negative effect on attention, working memory, and episodic recall. It has been shown that the effect of noise varies based on task complexity. For instance, studies have shown that noise has no effect on simple tasks but does play a factor in more complex tasks.^12^ Theories have been proposed to explain why there is a tendency for people to perform better in silence compared to an environment that contains background noise.^13^^,^^14^ One such theory is that background noise captures the individual’s attention and reallocates their attention away from the target information, causing an interruption in the task.^14^^,^^15^ However, prior research between the relationship of cognitive workload and noise stress has been ambiguous.^16^ It is well-established that the prefrontal cortex (PFC) plays a key role in higher order cognitive function,^17^ thus, the use of functional near-infrared spectroscopy (fNIRS) to measure workload is a natural step.

fNIRS is an optical method that measures the changes in concentration of oxy-hemoglobin (HbO) and deoxy-hemoglobin (HbD). fNIRS provides independent measures of the chromophores present in HbO and HbD by utilizing at least two different wavelengths that are differentially absorbed.^18^ fNIRS utilizes wavelengths within the 600- to 950-nm near-infrared region in tissues where scattering is the main photon transport mechanism.^19^ Active neurons attract more blood flood flow to their region as they require additional energy.^20^ Cortical neuronal activation increases cerebral oxygenation (neurovascular coupling) when specific stimuli are present, which make it possible to associate specific regions of the brain to cognitive functions.^21^ Previous studies have shown that by isolating the PFC, one can specifically look at mental workload.^22^ Another study revealed that when a person is stressed, the cortical activation in the PFC is reduced.^23^ Hemodynamic responses from the PFC have been shown to be a reliable measurement in complex naturalistic tasks in terms of quantifying cognitive workload levels.^24^ Using fNIRS to detect workload effects in the PFC, as a result of visual stimulation, has also been investigated.^25^ Ayaz et al.^26^ used fNIRS to detect the level of workload for participants piloting unmanned air vehicles. Solovey et al.^27^ performed a multitasking assignment to emulate real-world environments as opposed to the standard n-back task studies used to investigate mental workload.

In our study, we utilize fNIRS to address anomaly detection research by highlighting activation when anomalies are correctly detected and also missed while measuring performance under a variety of noise conditions. Behavioral data that include eye tracking, reaction time, and accuracy rate of target identification were also utilized in conjunction with fNIRS to aid in verifying the hemodynamic results. The study was undertaken specifically to quantify the effect of different noise stimuli on analysts’ performance and workload in anomaly detection by simulating a noisy work environment.

### 1.1 Significance

Missed anomalies can have a devastating effect on ISR operations as they can provide significant information for numerous applications.^28^ An understanding of how noisy work environments impact human analysts in anomaly detection could potentially lead to better performance by optimizing work environments. Most research in the past, when dealing with measuring workload in the brain, has been done with the use of electroencephalography (EEG) through monitoring brain activity. fNIRS has gained momentum as a brain imaging modality due to its ability to measure unique sets of physiological data (HbO and HbD) as well as lessen physical restrictions set on the user when compared to EEG systems.^29^ Previous studies have shown that situations of high cognitive workload correlated to decreased task performance in the field of anomaly detection.^16^ fNIRS has the capability of monitoring neural activation in the PFC, which has shown to correlate with workload. Therefore, by deploying fNIRS, quantifiable data can be obtained that can assess the effect of noise-filled work environments on human analysts. Our main objective was to perform fNIRS in adults participating in visual search tasks in various noise-filled environments to simulate the effect of a noisy work environment of an ISR analyst. We hypothesize that noisy environments would have a negative effect on the participant in terms of performance due to the increase in workload, which would be reflected by an increase in PFC activity. Therefore, we anticipate that significant HbO and HbD concentrations will arise in the PFC, following evidence from previous studies.^22^

## 2 Materials and Methods

### 2.1 Instrumentation

**Fig. 1:**
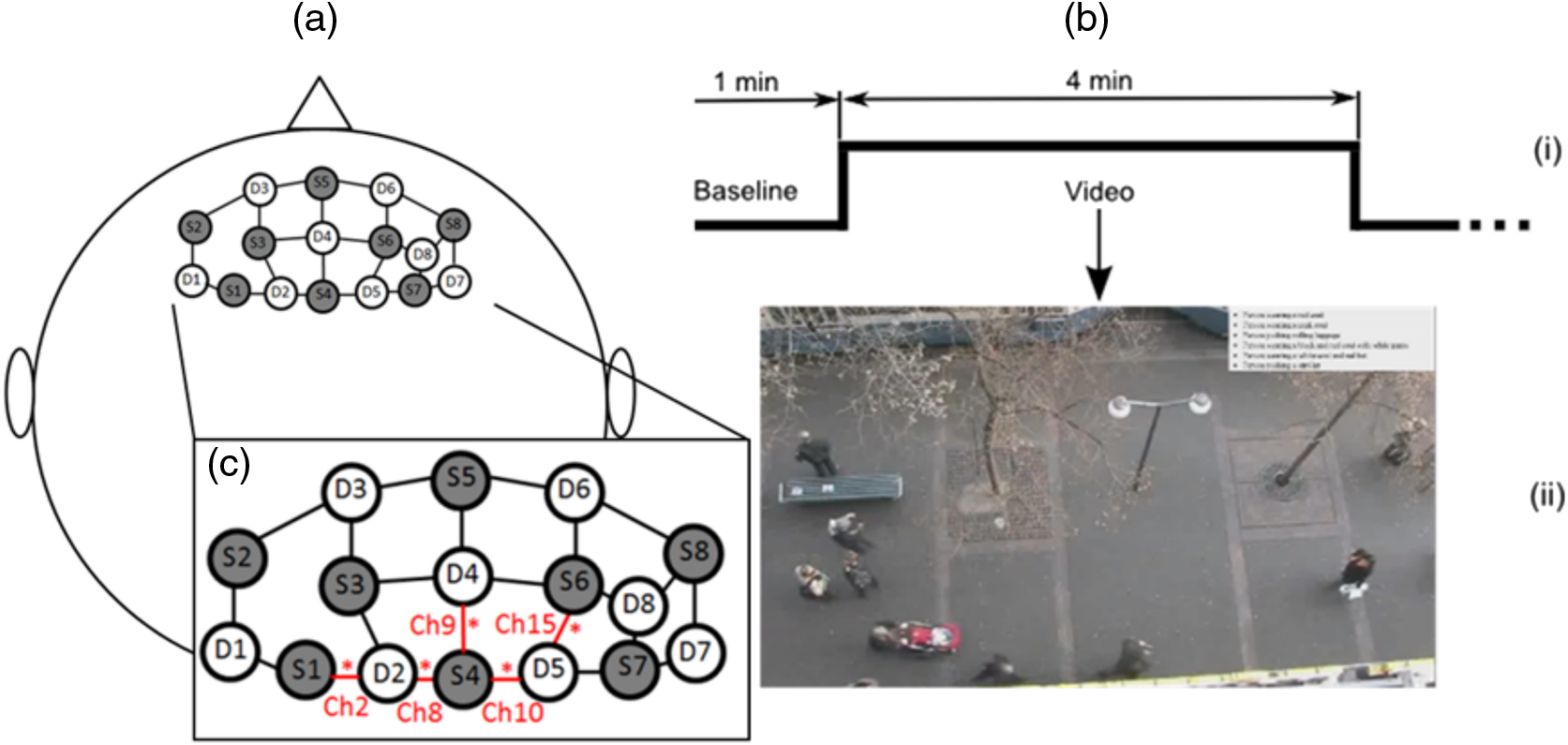
(a) Sources (black outline gray fill) and detectors (black outline white fill) layout based on 10–20 system to give a total of eight sources, eight detectors, and 23 channels. (b) Illustration of (i) task paradigm timing. A total of seven videos were used. (ii) Videos consisted of real-world scene with instruction to identify particular targets. (c) A zoomed-in view of the schematic shown in (a). Significant channels are indicated with asterisks and channel numbers. Channels 2, 9, and 15 were found to be significant for HbO. Channels 2, 8, 9, and 10 were significant for HbD.

A continuous wave, single phase, compact NIRScout imaging system (NIRx Medical Technologies LLC)—with eight 760- and 850-nm wavelength LED sources (power: >5  mW/wavelength) and eight Si photodiode detectors (sensitivity and dynamic range: <1  pW and 90 dB)—was used to measure changes in HbO and HbD at a sampling rate of 7.81 Hz. Changes in concentration of both HbO and HbD were defined as the differences between an average baseline and a stimulus-induced visual target. Data were recorded using NIRStar 14.2 (NIRx Medical Technologies LLC). With the aid of a retaining cap, all sources and detectors (optodes) were arranged to overlie the PFC (Fig. 1).

### 2.2 Experimental Protocol

The experiment was conducted at Wright State University (WSU) fNIRS Lab. The experiment consisted of seven different visual search tasks in the form of a live video feed that was obtained from Computer Vision Lab Walking Pedestrians dataset^30^ and were implemented in various environments through the use of different noise stimuli via headphones. Every video was ∼4  min in duration and contained six visual targets that consisted of four nonoverlapping and two overlapping targets. Examples of targets include a person pushing a stroller and a person wearing a red coat. The targets varied for each video by appearing at random time frames and were on screen for a variable amount of time. Therefore, each video was unique. The videos were randomized from subject to subject to eliminate any biasing, and a baseline of ∼1  min occurred prior to the start of each video (Fig. 1).

A textbox was present in the upper right-hand corner for the entire duration of each video that contained all of the targets in the order they would appear on the screen. The subjects were instructed to press key “4” on the keyboard when the specified target entered and key “6” when the target exited. Prior to the start of the experiment, the participants were given instructions and a demonstration via a practice video. The noise stimuli were presented through audio files that consisted of realistic environmental noises, such as a telephone ringing and a jet flying overhead. All of the noise stimuli were ∼70  dB. Videos varied based on duration of noise stimuli and/or time of introduction of noise stimuli. The experiment consisted of one factor (occupational noise) and four levels (type of noise). The types of noise included: no noise, short-intermittent noise-12 and -60, and long-intermittent noise. The experimental design for each video is shown in Table 1.

**Table 1:** Video presentations were the same for all conditions, but the type of distraction noise was varied. Design was divided into: no noise, short-60, long, and short-12.

Video	Type of noise	Onset of noise
1	No noise	No delay
2	Short-intermittent-60	5 s prior to target
3	Short-intermittent-60	No delay
4	Long intermittent	5 s prior to target
5	Long intermittent	No delay
6	Short-intermittent-12	No delay
7	Short-intermittent-12	5 s prior to target

The short-intermittent noise-12 stimuli consist of 12 noise occurrences that are each 2 s in duration while the short-intermittent noise-60 is composed of 60 2-s noises. The long-intermittent noise stimulus contains 12 noise occurrences that are each 10 s in duration.

In collaboration with fNIRS, eye tracking, reaction time, and accuracy rate were utilized in the experiment. The eye-tracking metrics include target fixation count rate and target fixation duration rate. The target fixation and duration rates are the number and duration of fixations, respectively, divided by the duration of the specific target when it comes on screen. By dividing fixation count and duration by the duration of the target, the data can be normalized between targets due to the targets differing from video to video in terms of the time they are present on the screen. The reaction time is composed of the target-in and target-out latency. The target-in latency refers to the time that the participant presses key “4” when they identify that the target is entering the screen minus the time the target actually comes into the video. The target-out latency is the time when the participant presses key “6” when the target exits the screen minus the actual time the target exits the screen. The accuracy rate is composed of the number of missed targets and false alarms. Missed targets refer to a target entering or exiting the video and the participants fail to press the corresponding computer key. False alarms refer to when the participant pressed key “4” or “6” when there is no target on the screen. The eye tracking, reaction time, and accuracy measures are displayed in Table 2.

**Table 2:** Dependent variables with measure details for eye tracking, reaction time, and accuracy metrics.

Dependent variable	Measure
Target fixation count rate (per unit time)	(Number of target fixations)/(duration of target on screen)
Target fixation duration rate (per unit time)	(Target fixation duration)/(duration of target on screen)
Target-in latency	(Key “4” pressed time) – (duration of target when it comes on screen)
Target-out latency	(Key “6” pressed time) – (duration of target when it comes on screen)
Missed targets	Number of missed targets throughout the video
False alarms	Number of false alarms when pressing the “4” or “6” keys

### 2.3 Participant and Preparation

A total of 10 participants [38.30±4.61  years, mean±standard error (SE)] were recruited at WSU. Three were female (38.00±4.91  years) and seven were male (38.43±4.89  years), and all were right-hand dominant. The experimental protocol was approved by the institutional review board at WSU, and informed consent was obtained from each participant prior to involvement in the study.

Subjects were seated in front of a Tobii T120 Eye Tracker monitor with a screen size of 17 in. and resolution of 1280×1024  pixels. After an optode retaining cap was placed on the subject’s head and centered, clear saline electrode gel was applied to enhance the signal quality by keeping the hair aside and improving optode contact with the skin. The gel application process allowed hair to be pushed and kept aside before inserting optodes into the cap. Measurements were taken in a quiet and darkened room. A large, fleece cap was placed over the subject’s head to further reduce any outside light that could possibly strike the detectors. Each participant was asked to focus on the eye-tracking monitor during data collection while remaining silent and as still as possible, aside from the hand-movement tasks associated with identifying the visual targets. Participants that had increased movement during the experiment were well-documented to correct for the motion during the preprocessing steps of data analysis.

### 2.4 Data Analysis

Raw data were processed using the functions of Homer2^31^–MATLAB^®^-based (The Mathworks, Inc.) analysis tool and used to extract relevant values, such as target and baseline concentrations. A 0.01- to 0.1-Hz bandpass filter was used, which allowed the elimination of low-frequency system drift and physiological noise, such as heart rate, without removing artifacts that may be stimulus induced. Filtered signals were translated to changes of hemoglobin concentration using the modified Beer–Lambert law. Differential pathlength factor values for this region and a mean age of 38.3 years were estimated to be 6.14 and 5.09 for wavelengths of 760 and 850 nm, respectively, based upon calculations from previous studies.^32^^,^^33^ The filtered signals were visually inspected, and noisy data were removed to mitigate the effect of any motion artifacts that were not removed by the bandpass filter.

### 2.5 Statistical Analysis

Quantitative statistical analysis of the mean hemoglobin value of the time points between the onset and exit of each respective nonoverlapping target subtracted by the mean of each target’s baseline was performed on all the optode channels. Subject heterogeneity and head sizes vary, and therefore recordings may not always come from the same specific region of interest. Here, baseline is defined as the average concentration during the 5-s period before each target. Mixed effects models were used to account for within-subject variability and repeated measures. The type of noise levels were compared for each individual channel. This was performed on the group levels, and all tests were two-sided and performed at the significance level (α)=0.05. JMP 11^®^ by SAS^®^ (SAS Institute Inc., 2013, Cary, North Carolina: SAS Institute Inc.) was used to perform statistical tests. All values are written as mean±SE.

## 3 Results

**Fig. 2:**
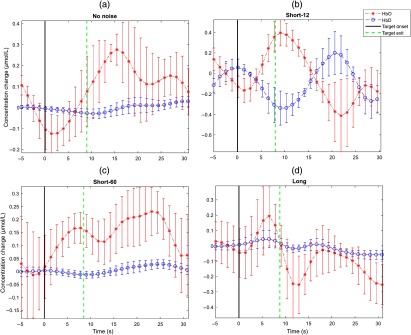
An illustration of the block-averaged hemodynamic responses for a target response across all the participants and noise types. The error bars represent SE. HbO curves are asterisk lines and HbD curves are circular lines. The solid vertical line indicates the onset of the target. The dashed vertical line indicates the exit of the target. (a) Represents the no noise, (b) is the short-12, (c) is the short-60, and (d) is the long noise stimulus.

From the 10 subjects that were tested, a total of eight subjects were used in the analysis as two subjects were excluded due to fNIRS system failure and noise-filled signals. After removing outliers and noisy data, the hemodynamic responses for both HbO and HbD were calculated for all of the channels (1 to 23). A representation of the block-averaged hemodynamic responses for a target response for all the participants and type of noise levels are shown in Fig. 2.

**Fig. 3:**
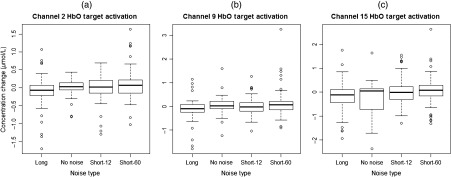
Mean HbO concentration changes (μmol/L) for significant channels across the different noise types. (a) The long noise level is significantly lower than the short-60 condition for HbO in channel 2. (b) In channel 9 for HbO, the long noise stimulus is significantly lower than the short-60 level. (c) The short-60 is significantly higher compared to both the long and no noise levels for HbO in channel 15.

From the 23 channels that were analyzed, there were three statistically significant channels for HbO (channels 2, 9, and 15) and four significant channels (channels 2, 8, 9, and 10) for HbD. The significant channels are illustrated in Fig. 1. For HbO in channel 2, the long-intermittent noise (mean=−0.11, SE=0.05  μmol/L) was significantly lower compared to short-60 (0.089±0.05  μmol/L, p-value=0.026), but there were no significant differences between the long, no noise (−0.016±0.07  μmol/L), and short-12 (−0.019±0.05  μmol/L) levels. The long noise level (−0.12±0.06  μmol/L) for HbO in channel 9 was significantly lower than short-60 (0.15±0.06  μmol/L, p-value=0.022), but the long noise was not significantly different in comparison to the no noise (0.063±0.09  μmol/L) and short-12 (−0.018±0.06  μmol/L) levels. In channel 15 for HbO, short-60 (0.098±0.08  μmol/L, p-value=0.019) was significantly higher in comparison to both the no noise (−0.24±0.12  μmol/L) and long (−0.17±0.08  μmol/L) levels, but there were no statistical differences between the short-60 and short-12 (0.018±0.08  μmol/L) noise stimuli. The box and whisker plots for the significant HbO channels are shown in Fig. 3.

**Fig. 4:**
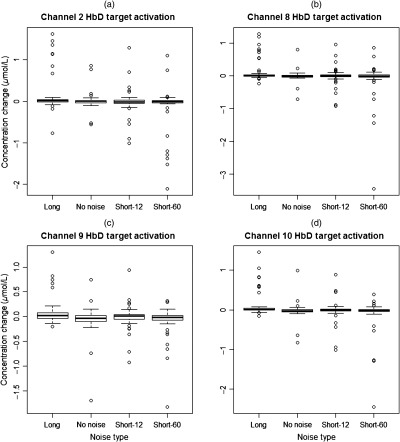
Mean HbD concentration changes (μmol/L) for significant channels across the different noise types. (a) The long level is significantly higher in comparison to both the short-12 and short-60 levels in channel 2 for HbD. (b) In channel 8 for HbD, the long noise is significantly higher compared to the short-60 level. (c) The long noise is significantly higher compared to both the no noise and short-60 levels for HbD in channel 9. (d) In HbD for channel 10, the long level was significantly higher compared to both the short-12 and short-60 stimuli.

For HbD in channel 2, the long-intermittent noise (0.12±0.05  μmol/L, p-value=0.0060) was significantly larger in comparison to both the short-12 (−0.029±0.05  μmol/L) and short-60 (−0.11±0.05  μmol/L) levels but not statistically different than the no noise condition (0.020±0.07  μmol/L). For HbD in channel 8, the long noise (0.10±0.04  μmol/L, p-value=0.0082) was significantly larger than the short-60 condition (−0.095±0.04  μmol/L). There were no statistical differences between the long, no noise (−0.0089±0.06  μmol/L), and short-12 (−0.011±0.04  μmol/L) levels. The long-intermittent noise (0.077±0.03  μmol/L, p-value=0.0014) for HbD in channel 9 was significantly larger in comparison to both the no noise (−0.072±0.05  μmol/L) and short-60 (−0.080±0.0  μmol/L) levels. There were no statistical differences between the long and short-12 (−0.011±0.03  μmol/L) noise stimuli. In channel 10 for HbD, the long noise stimulus (0.10±0.04  μmol/L, p-value=0.0016) was significantly larger than both the short-12 (−0.016±0.04  μmol/L) and short-60 (−0.088±0.04  μmol/L) noise levels. There were no statistical differences between the long and no noise (−0.022±0.05  μmol/L) conditions. The box and whisker plots for the significant HbD channels are shown in Fig. 4.

Target-in and target-out latency was analyzed via paired student’s t-test, and there was no statistical significance between the noise levels. However, short-12 had the highest mean for both target-in and target-out latency (6.57±0.90 and 4.16±0.97, respectively) while short-60 (5.49±0.90 and 2.52±0.97, respectively) had the lowest. There was also no statistical difference for both the false alarm rate and missed targets. However, short-12 (2.63±0.47) and short-60 (1.69±0.47) had higher mean false alarms compared to the long (1.38±0.47) and no noise (1.13±0.67) levels. For the missed targets, the short-12 (1.63±0.41) and no noise (1.50±0.58) levels had the highest average while the long (1.44±0.41) and short-60 (mean=1.06±0.41) had the lowest.

Target fixation count rate and target fixation duration rate had statistical significance. In both cases, the mean of the long noise (1.12±0.05 and 0.11±0.005, respectively) was the greatest compared to the other levels, and short-12 (0.92±0.05 and 0.070±0.005, respectively) was the lowest. For the fixation count rate, the long-intermittent noise (p-value=0.0047) was significantly different than both the short-12 (p-value=0.045) and no noise levels. For the target fixation duration rate, the long noise (p-value<0.0001) was statistically different than the short-60, no noise, and short-12 (p-value<0.0001) levels.

## 4 Discussion and Conclusion

This study aimed to assess the fNIRS signal activation at the PFC in adults during visual search tasks in various noise-filled environments to simulate the workload of an ISR analyst. The salient findings of our study both partially support and go against our hypothesis that PFC activity would increase as a result of a noise stimulus during a visual target search task. The findings partially support our hypothesis as a result of HbO activation in channel 15 for short-60 being significantly higher in comparison to the no noise condition, hence indicating that this noise condition causes an increase in cognitive workload. The findings go against our hypothesis as a result of the no noise condition having a higher PFC activation compared to the long noise in channel 9 for HbD. Also, there were no significant differences between the no noise, long, and short-12 levels for HbO. This could be due to increased workload of the noise stimulus causing blood flow to be redirected to other portions of the brain, thus reducing the amount of blood flow to the PFC leading to no statistical difference for noise versus no noise. During the periods of multitasking, load sensitive brain regions can elicit either transient or sustained activation, which suggests that these regions could be involved in time-constrained activities such as memory updating. According to Wickens et al.,^34^ “the resources on which this updating activity depends seem to be limited in their availability, and, when deployed in the service of one task, their availability to be of service to other tasks is reduced.” Due to the temporal aspect of these activations during multitasking, some of these neural stimulations could explain the decline in stimulation in one region (specifically, PFC) even though this region could be heavily involved. On the same note, increasing load on working memory and workload does not always result in a decrease in performance,^35^ which could also explain why there were no significant findings with the reaction time and performance measures.

However, there were significant differences for HbO activation in channels 2, 9, and 15 due to short-60 being significantly higher in comparison to the long noise stimulus. Also, both short-12 and short-60 were found to have a significantly higher neural activation compared to the long noise stimulus in channels 2 and 10 for HbD. Therefore, the short-intermittent noises could mimic “alarms” that distract the participants attention away from their primary task (target identification) while the long noise is more continuous and could serve as “typical background noise” that the participants experience in their daily life. Furthermore, previous studies have indicated that HbO activation due to continuous noise is significantly lower than short-intermittent noise in regions of the PFC.^36^^–^^39^ This implies that workload and working memory could be adversely affected by pulsated noises as these distractions can result in increased neuronal activation as it takes a larger cognitive effort to perform the task at hand. Pulsated noise also activates auditory regions of the brain to a larger degree compared to more continuous noises.^39^^,^^40^ Therefore, pulsated noise habituates to a lesser degree compared to continuous noise due to it containing more information.^39^

Stressors, such as time and noise, also play a factor in anomaly detection due to their influence on attention, executive function, and memory. Prior research has been conducted to assess the relationship between noise exposure and cognitive performance; however, the results have been ambiguous, as the effects of noise on performance have been found to be facilitative, detrimental, or even absent.^16^ Research has also shown that situations of high cognitive load correlate to increased error rates in anomaly detection by the human analyst.^16^ The target fixation duration rate, another indicator of cognitive workload, resulted in a significantly higher mean for the long-intermittent noise compared to the other levels. This suggests that the participants were more focused on the targets during the long noise as they entered and exited the screen. A previous study found that a decrease in target fixation rates could be indicative of “visual tunneling” on nontarget stimuli due to the participants having a reduced range of scanning. This reduced range of scanning could be the outcome of increased workload.^41^ This increased workload can account for the significant difference in HbO and HbD activation in the short-noise setting compared to the long noise.

With regards to fNIRS, the PFC serves a vital role as it is related to memory tasks.^42^ It is believed that the PFC promotes mental manipulations with regards to the central executive of working memory. Studying the PFC during periods of high working memory-intensive tasks is important because during this time, there is a change in the regional cerebral blood flow. This would indicate an increase in neuronal activation, in which fNIRS measures in the form of oxy-Hb concentration.^43^ Herff et al.^42^ showed that fNIRS signals obtained from the PFC are indicators that can be utilized to quantify user workload. Bendall et al.^44^ also demonstrated that NIRS is a well-suited technique to measure PFC activity during cognitive tasks. Specifically, previous fMRI studies have demonstrated that the posterior medial and dorsolateral PFCs are active in both working memory and workload tasks.^45^^–^^48^ The posterior medial PFC has also been shown to play a key role in decision-making.^48^^,^^49^ The significant channels in this study (Fig. 1) correspond to the previous findings as they are mainly congregated around the posterior medial PFC.

Overall, fNIRS is a promising domain, which can have major impacts in terms of military applications. Specifically, being able to “quantify” mental workload does not only impact anomaly detection but can play a huge role in influencing the probability of human error during remotely operated vehicle (ROV) operations, for example, where safety hinges on the operator’s ability to make instantaneous decisions.^50^ A high workload can result in the operator making rash and ill-advised decisions, which can have catastrophic outcomes. According to the Office of Aerospace Medicine in the United States, human factors-related deficiencies are responsible for 21% to 67% of ROV accidents in the US Army, Navy, and Air Force.^51^ These are preventable, and fNIRS can play a major role in helping to bring these numbers down. By analyzing the effect of workload and environmental noise stressors via the utilization of fNIRS technology, the optimal environment for the ROV operators can be determined. This can possibly lead to an increase in work efficiency and a decrease in the number of human factor-related accidents.

Like any experiment, our study has several limitations. This study only investigated HbO and HbD activation in the PFC region; therefore, we were unable to measure other regions of the brain, such as the motor, visual or auditory cortices, which could experience significant changes in cerebral blood flow. Cap configurations for the former two cortices have been tested and optimized.^52^^,^^53^ Signals from the motor cortex as a result of hand movements could be used as a regressor to clean up the data attained from the PFC. Correlation of the visual and auditory cortices with the PFC could further elucidate cognitive workload findings. Live video feed was used as the visual stimuli to simulate an ISR analyst task that could make one of the seven videos that the participant faced more challenging or easier than the others that could alter our results. fNIRS is unable to measure deep-brain regions due to experiencing limited sensitivity to hemodynamic changes in these areas.^54^ Therefore, fNIRS can only measure cortical regions of the brain.^55^ Additional fNIRS limitations include interferences from nontargeted chromophores (HbO and HbD), indefinite differential pathlength, and unknown scattering loss factor.^56^ Future work will consist of investigating the overall trend of HbO and HbD during overlapping targets.

In conclusion, fNIRS has been shown to measure significant differences in both HbO and HbD activation in the PFC cortex between short- and long-intermittent noises during a visual search task. This suggests a difference in workload or working memory under different noise stimuli as certain regions of the brain could be heavily involved for the processing of one type of noise due to it acting like a distraction but not so much for another noise type. A more comprehensive study, including different noise amplitude and noise frequency, could lead to significant results in performance measures that could further explain the impact of noise on anomaly detection.
